# Exploring the link between anticipatory outcome encoding in the brain and goal-directed behavior during outcome devaluation

**DOI:** 10.1162/IMAG.a.956

**Published:** 2025-10-24

**Authors:** Jasmin Stein, Hannes Ruge, Uta Wolfensteller, Thomas Goschke, Katharina Zwosta

**Affiliations:** Faculty of Psychology, TU Dresden, Dresden, Germany

**Keywords:** goal-directed behavior, fMRI, MVPA, outcome devaluation, dlPFC

## Abstract

Goal-directed behavior is thought to rely critically on the anticipation of potential future outcomes of an action. In this study, we used an fMRI instrumental learning paradigm with selective outcome devaluation in a sample of 59 participants (ages 18–33) to identify regions of the brain’s goal-directed system showing anticipatory neural representations of action outcomes. Using multivariate pattern analysis, we could show that an area in the left dorsolateral prefrontal cortex significantly encoded action outcomes in an anticipatory manner. Critically, anticipatory outcome encoding strength in a subset of voxels in this dorsolateral prefrontal cluster significantly predicted behavioral sensitivity to outcome devaluation, hence behavioral goal-directedness. This finding is important because anticipatory outcome encoding in dorsolateral prefrontal cortex (dlPFC) has never been directly linked to behavioral goal-directedness during outcome devaluation in previous research. However, the finding presented here is preliminary and needs to be replicated systematically. In addition, future research is needed to investigate the specific role of different regions along the lateral prefrontal cortex in this context and to investigate whether the effect reported here can explain impairments in goal-directed behavior under specific conditions such as, for example, the experience of acute stress.

## 1 Introduction

Human behavior is assumed to rely on two distinct brain systems: a goal-directed system enabling flexible adjustment of actions to ever-changing environmental conditions and anticipated outcomes of our actions, and a habitual system relying on stimulus-response associations promoting fast and stimulus-driven behavior (Balleine & Dickinson, 1998). While the goal-directed system is necessary for flexible decision-making in changing environments, flexibility comes at the cost of higher cognitive demand and longer processing times (Dolan & Dayan, 2013). Thus, when time is of the essence, relying on the habitual system might yield a critical advantage (at the cost of behavioral flexibility, however). Given the (dis)advantages associated with either system, the flexible use of both systems is an important prerequisite for adaptive behavioral control. Indeed, it has been demonstrated that both, habitual and goal-directed processes, can be present simultaneously (Luque et al., 2017) and there is evidence for both processes to be independent of each other (Meier et al., 2022). Therefore, the specifics of their interplay represent an active field of research.

A core aspect of goal-directed behavioral control is the anticipation of future outcomes of our actions, hence forming a causal association between behavioral responses (R) and their respective outcomes (O) (Balleine, 2019; O’Doherty et al., 2017). Habitual behavior, on the other hand, relies on forming associations between stimuli (S) and behavioral responses (R) and thus reflects stimulus-driven, automatic behavior (Dolan & Dayan, 2013). The notion that outcome anticipation is a key element of volitional action control is pervasive across influential theoretical approaches such as instrumental learning theories (Balleine & Dickinson, 1998), ideomotor theories (Elsner & Hommel, 2001; Hommel, 2009, 2019), reinforcement learning (Daw et al., 2011), or integrative computational models of adaptive behavior (Soltani & Koechlin, 2022).

In human research, a variety of experimental designs have been implemented to study the distinction between goal-directed and habitual behavior (e.g., De Houwer et al., 2018; de Wit et al., 2012; Luque et al., 2017; Sjoerds et al., 2016; Tricomi et al., 2009; Watson et al., 2023; Zwosta et al., 2018). The rationale of these studies is that habitual S-R associations cause measurable erroneous behavior when dynamic changes in the task at hand require a behavioral response that differs from an established habit. For example, in so-called outcome devaluation tasks (e.g., Tricomi et al., 2009), one specific action outcome is selectively devalued after a period of instrumental contingency learning. If participants were to act goal-directed, so the assumption, they should adjust their behavior to anticipated changes in outcome value, for example, by responding considerably less in trials where a devalued outcome would be anticipated following a response. Habitual behavior, on the other hand, should, independent of changes in outcome values, lead to continued response behavior in line with the previously learned contingencies. Note that, while devaluation paradigms are often used to delineate neural correlates of goal-directed and habitual behavior, the focus of this study was to identify neural outcome anticipation processes supporting goal-directed behavior and not on habitual behavior or its neural basis.

FMRI studies have identified brain regions playing a critical role in encoding outcome anticipations or integrated response-outcome contingencies in humans. An important candidate region is the ventromedial prefrontal cortex (vmPFC) which was reported to encode action-outcome contingencies (e.g., Liljeholm et al., 2011; Morris et al., 2014; Tanaka et al., 2008) and anticipated outcomes prior to outcome presentation (e.g., McNamee et al., 2015). A further cortical region reported to be implicated in anticipatory outcome encoding is the dorsolateral prefrontal cortex (dlPFC; McNamee et al., 2015; note, however, that the ROI used by McNamee et al., 2015, lies more posterior to regions typically labeled as dlPFC and will be referred to as posterior LPFC in this work). In addition, activation strength in and structural connectivity of striatal seeds with the dlPFC have been linked to successful behavioral adjustment to outcome devaluation (van Timmeren et al., 2024; Watson et al., 2018). Finally, the angular gyrus was also implicated previously in outcome-based decision-making (e.g., Zwosta et al., 2015, 2018). Subcortically, the (anterior) caudate nucleus plays an important role in action-outcome learning and is hence considered part of the goal-directed brain system in humans (Balleine & O’Doherty, 2010; Morris et al., 2022; Tanaka et al., 2008; Tricomi et al., 2004) while the putamen, on the other hand, has been suggested to be part of the habitual brain system (Balleine & O’Doherty, 2010; Tricomi et al., 2009). Further, the interplay of these regions in cortico-striatal networks is pivotal to the expression of goal-directed and habitual behavior (de Wit et al., 2012; Ruge & Wolfensteller, 2013, 2015; Seger, 2018; van Timmeren et al., 2024). Beyond the aforementioned circuits, lower-level perceptual brain areas (e.g., areas along the ventral visual stream) are involved in anticipatory outcome identity encoding when outcomes are represented by perceptual stimuli, for instance, images of faces, objects, or scenes; see Waszak et al. (2012) for a brief review.

While many studies have successfully identified regions of the goal-directed brain circuit in humans, the investigation of anticipatory outcome representations and their role in goal-directed behavior is still ongoing. Several fMRI studies have used multivariate analysis methods to study anticipatory neural representations of important aspects of goal-directed behavior (Howard & Kahnt, 2017; McNamee et al., 2015; Meier et al., 2022; van Steenbergen et al., 2017; Wisniewski et al., 2019; Yan et al., 2016). The rationale of these studies is often to identify brain areas that encode response-associated outcome identities or outcome values in an anticipatory fashion, explicitly during time windows before an outcome or associated reward is indeed presented. This methodology allows researchers to specifically investigate the anticipatory nature of response-outcome associations. Of specific note is a study by Meier et al. (2022) linking neural signatures of outcome anticipation directly to behavioral performance in an outcome devaluation paradigm. The authors used EEG and an outcome devaluation paradigm to show that stress-induced changes in anticipatory neural outcome representations over time were significantly correlated with behavioral sensitivity to devalued outcomes such that participants with decreasing outcome representations over time were less sensitive to selective outcome devaluation. However, due to the limited spatial resolution of EEG, the precise neural circuits underlying the association between anticipatory outcome encoding and goal-directed behavioral control remain to be investigated. In this study, we aimed to fill this gap by combining fMRI with an outcome devaluation paradigm and the use of multivariate analysis techniques to identify brain areas involved in anticipatory outcome encoding and establish a link between outcome anticipation and behavioral goal-directedness.

Specifically, we aimed to test the following hypotheses: 1) Regions of the goal-directed brain system, specifically the dlPFC, vmPFC, anterior caudate, and angular gyrus, show significant anticipatory encoding of outcome identities; 2) The individual strength of the identified anticipatory representations will be significantly correlated with behavioral goal-directedness such that higher encoding strength is associated with more flexible behavioral adjustment to selective outcome devaluation.

## 2 Materials and Methods

This study was approved by the Ethics Committee at TU Dresden, Germany (EK580122019). Informed consent was obtained from all participants for being included in the study.

### 2.1 Sample

Initially, 70 participants completed the full experimental session. After a quality assessment of the collected data, 11 participants were excluded (five due to an initial error in the experimental code, one due to clinically relevant abnormalities in the MRI scan, one due to excessive head motion peaks of more than 1.5 cm framewise displacement (FD) in each run, one due to technical issues with the scanning procedure, and three participants who failed to correctly report the learned outcomes associated with each stimulus after having completed the full experiment). Consequently, we included 59 participants in our analysis sample (39 female and 20 male; mean age: 23.44 years, SD=4.01 , largely university students). For all analyses investigating devaluation block trials, two additional participants had to be excluded since they largely failed to respond during trials with devalued outcomes (13 and 15 trials out of 16 trials, respectively, with missing answers) likely indicating a lack of understanding of the instruction to press the alternative button when anticipating a devalued outcome.

We recruited participants via an online recruitment system at TU Dresden and flyers and posters across the university campus and the city of Dresden. All participants were right-handed German native speakers between the ages of 18 and 35. Further, we only included participants without current diagnoses of psychiatric or neurological disorders who did not report frequent smoking (<5 cigarettes per week) or drug consumption. Further requirements for study participation were normal or corrected-to-normal visual acuity and fulfilling standard MRI safety criteria. All participants provided informed consent at the beginning of the study and were reimbursed with 10 Euros/hour (30 Euros for the full experimental session) or course credit. In addition, participants received additional financial compensation based on their task performance.

### 2.2 Data collection procedure

**Fig. 1.**
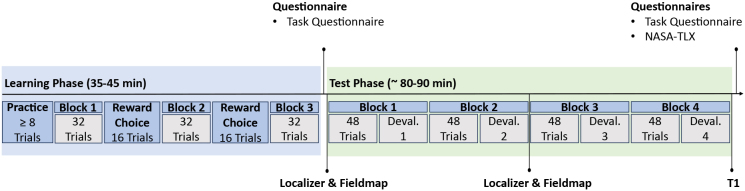
Experimental timeline including a learning phase outside the MRI scanner, a test phase containing two experimental runs consisting of two blocks each in the MRI scanner, and the completion of questionnaires. The full experimental session lasted around 3 h.

**Fig. 2.**
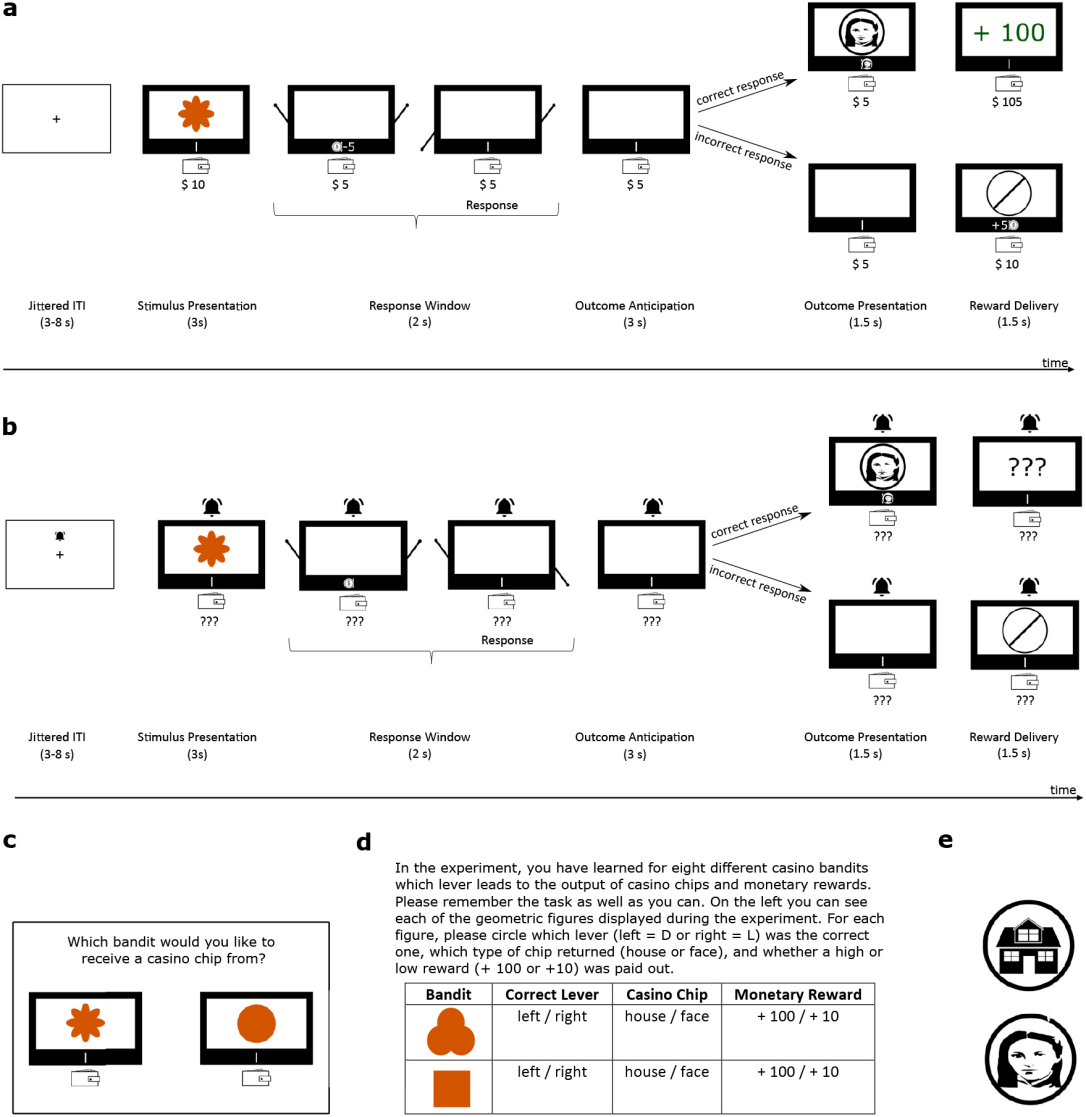
Important elements of the experimental procedure. (a) Trial timeline of regular trials. Each regular trial started with the presentation of a stimulus followed by a response window. Upon a correct answer (top), an outcome was presented after an outcome anticipation phase. Finally, a reward was delivered. In case of an incorrect response (bottom), no outcome was presented and a crossed-out circle during the reward delivery phase provided error feedback. In addition, the response cost was returned to the participant’s wallet. The ITI was jittered between 3 and 8 s; (b) Devaluation trials. Devaluation trials followed the structure of regular trials, except for the presentation of an alarm bell and the masking of all rewards; (c) Exemplary depiction of a reward choice trial; (d) Exemplary depiction of two (out of eight) items of the task questionnaire; (e) House and face stimulus used as outcomes.

All participants were screened for inclusion criteria. The screening was conducted via telephone. Upon successful inclusion, participants were invited to the laboratory session. Here, participants first provided informed consent. Subsequently, all participants completed the learning phase of the experiment (see Section 2.3.1). After completion, participants were asked to fill out a questionnaire assessing explicit knowledge of task contingencies (custom questionnaire where all eight experimental stimuli were depicted and participants had to indicate for each stimulus the corresponding correct response, outcome, and reward magnitude; see Fig. 2d for a depiction of the questionnaire). Afterward, participants were placed into the MRI scanner to complete the test phase of the experimental paradigm (see Section 2.3.2). Upon completion of the scan, participants were removed from the scanner and completed the task questionnaire again. Participants also completed the NASA-TLX questionnaire (Hart, 2006) to assess experienced task load. Finally, all participants were reimbursed for their participation and the points collected throughout the experiment. The laboratory session had a full duration of around 3 h. The experimental timeline is shown in Figure 1.

### 2.3 Experimental paradigm

We developed an instrumental learning paradigm including selective outcome devaluation phases based on similar experiments from past research (Luque et al., 2017; McNamee et al., 2015; Meier et al., 2022).

In brief, we employed an instrumental learning paradigm where participants were instructed to learn eight different stimulus (S)-response (R)-outcome (O)-reward (Rew) contingencies. The experiment contained eight different stimuli (geometric shapes depicted in orange color). The stimuli were presented at the center of a two-arm casino bandit presented to the participants in each trial (see Fig. 2a for a visualization). Each stimulus was associated with one correct response (left or right key, corresponding to a virtual pull of the left or right lever of the casino bandit), one outcome (house or face, corresponding to a casino chip returned by the bandit depicting a house or face image), and one reward magnitude (low or high: 10 or 100, corresponding to the payout of virtual casino Dollars). As such, each of eight unique R-O-Rew combinations was associated with one specific stimulus. Importantly, by choosing eight different stimuli, we made sure that responses, outcomes, and rewards were orthogonalized with respect to each other. For an example of S-R-O-Rew combinations see Table 1. The association between stimuli and R-O-Rew contingencies was pseudorandomized across participants: We created eight different randomization patterns (one full rotation of the association between stimuli and R-O-Rew contingencies). Each participant was assigned one of these randomizations pseudorandomly; note that the randomization sequences occurred roughly equal in the final sample.

**Table 1.** Exemplary stimulus-response-outcome-reward combinations.

Stimulus	Response	Outcome	Reward
1	left	house	+ 100
1	right	-	-
2	left	-	-
2	right	face	+ 100
3	left	house	+ 10
3	right	-	-
4	left	-	-
4	right	face	+ 10
5	left	face	+ 100
5	right	-	-
6	left	-	-
6	right	house	+ 100
7	left	face	+ 10
7	right	-	-
8	left	-	-
8	right	house	+ 10

In each regular trial (see Fig. 2a for visual details), participants were presented with a two-arm casino bandit. The bandit displayed one of eight different stimuli (different geometric shapes presented in orange color, see Supplementary Fig. S1) for 3 s. Subsequently, the stimulus disappeared, and the two arms appeared on the left and right side of the bandit initiating a response window. At the same time, 5 virtual Dollars from the participant’s wallet were inserted into the bandit constituting a response cost that was applied in every trial. During the 2-s response window, participants could pull a lever of the bandit by pressing a left or right response button using their respective index finger. Upon a button press, the corresponding arm of the bandit was lowered on the screen and remained in this position throughout the rest of the 2-s response window. The response window was followed by a 3-s outcome anticipation window. During this time frame, the bandit’s arms disappeared, and the bandit was displayed blankly. Upon a correct response, the anticipation phase was followed by the presentation of a response outcome. Depending on the presented stimulus, a casino chip depicting an image of a house or a face (see Fig. 2e for a depiction of the house and face we used) was displayed. This outcome presentation lasted 1.5 s and was followed by the presentation of a reward. During the reward presentation phase, the bandit displayed either ‘+ 10’ or ‘+ 100’ depending on the given S-R-O-Rew contingency in each trial. The reward phase was presented for 1.5 s. In case of an incorrect response, the bandit remained blank during the outcome presentation phase. During the reward presentation phase, a crossed-out circle provided error feedback. Here, the 5 Dollars inserted as the response cost were returned to the participant. In trials where participants failed to indicate a response in time, the response cost was not returned. This was implemented as an incentive for participants to engage in the task and to encourage active response behavior in devaluation trials (see Section 2.3.2). Additionally, the feedback ‘Too slow!’ was presented if participants failed to provide an answer, and ‘+ 0’ was depicted next to the coin slot. An example of a correctly and an incorrectly answered trial is depicted in Figure 2a. The inter-trial interval (ITI) was jittered as an exponential between 3 and 8 s. During the ITI, a fixation cross was presented at the center of the screen.

Note that we chose house and face outcome stimuli since these are encoded along the ventral visual stream in a category-selective manner (Ishai et al., 2000) and have been used previously in the outcome anticipation literature (see, e.g., Waszak et al., 2012, for a brief overview). Monetary rewards were added in addition to visual outcomes for two reasons: first, to incentivize participants to learn the contingencies and provide correct responses, and second, to be able to conduct the devaluation procedure and apply response costs which were crucial for the devaluation manipulation (see Section 2.3.2).

#### 2.3.1 Learning phase

In the learning phase, participants practiced the experiment outside the MRI scanner. Participants were instructed that they would play for money in a virtual casino. They were told that they would be presented with eight different bandits, each characterized by a unique geometric shape. Participants were instructed to wait until the bandit’s arms turned visible in each trial and then to press the left (D) or right (L) key on the keyboard with the index finger of the corresponding hand. Participants were further instructed that a correct response would lead to the delivery of a casino chip with a house or a face depicted on it and consequently to the delivery of a reward of 10 or 100 fictional Dollars. Participants were also instructed about the nature of the response cost. Then, participants completed practice trials. The practice trials comprised the presentation of each of the eight bandits in fixed order. Each participant started this task with 10 virtual Dollars in their wallet. In case of a correct response, the next stimulus was presented. In case of an incorrect response, the same stimulus was repeated until a correct response was given. As soon as a correct response to all eight stimuli was given once, the instructions continued. Participants were instructed to remember which stimulus was associated with which casino chip (house or face) and which reward magnitude (10 or 100). They were told that this information would be relevant later on during the experiment because of the occurence of devaluation trials (see Section 2.3.2). Additionally, participants were told that in some trials, they would be presented with two bandits. Here, they could choose which bandit they wanted to receive the corresponding casino chip and associated reward from (reward choice trials, see below). They were told that the best strategy was thus to remember which bandits were associated with the higher or lower reward and make their choice accordingly. Finally, participants were instructed that the amount of virtual Dollars gained during the experiment would enhance their actual reimbursement at the end of the study.

After these instructions, participants completed three experimental blocks containing 32 regular trials each (four presentations of each stimulus in fully randomized order within each block). After the first two blocks, participants completed one reward choice block each. Each reward choice block consisted of 16 trials in which participants made choices between one bandit associated with a high and one bandit associated with a low reward magnitude. Each bandit was presented on one side of the screen (see Fig. 2c for a visualization), and participants made their choices using the 8 and 9 keys on the keyboard with the index and middle fingers of their right hand. Upon choosing one of the bandits (no time limit was employed, answering was required to continue the experiment), the outcome and reward associated with the bandit were shown sequentially at the center of the screen for 1.5 s each, and the reward was added to the participant’s wallet. The reward choice blocks contained all 16 unique combinations of the four high and low reward bandits in one spatial configuration in the first and in the reverse spatial configuration in the second reward choice block in fully randomized order.

Upon completion of the learning phase, participants were given feedback about their response accuracy and the amount of payout that they gained during the learning phase. To calculate this sum, the amount of virtual Dollars gained throughout the learning phase was converted to Euros (with roughly every 1200 virtual Dollars yielding an additional payout of 50 cents) such that payouts were made in steps of 50 cents and the maximally achievable sum was capped at a reasonable value (10 Euros for the full experiment). Note that this information was not explicitly disclosed to the participants. The learning phase took around 35 to 45 min.

#### 2.3.2 Test phase

The test phase took place in the MRI scanner. Before starting, participants were briefly reminded of the task. In addition, participants received instructions about the devaluation blocks and the included consumption trials occurring throughout those blocks. Specifically, the participants were told that in some blocks of the experiment, counterfeit casino chips (either houses or faces) were circulating in the casino and that the casino would hence not exchange chips depicting houses or faces (depending on the devaluation block) for money in this block anymore. They were also instructed about the best strategy in these devaluation trials: If encountering a bandit that would return the non-devalued type of casino chip upon making a correct response, participants should continue as practiced and strive for a correct response. However, if encountering a bandit that would return the devalued type of casino chip, it was the best strategy to select the originally incorrect answer. That is because a correct answer would lead to the return of the devalued chip and hence no reward delivery, while the 5-Dollar response cost would still be subtracted from the participant’s wallet. Providing no answer at all would lead to the same effect. Only pressing the key constituting an incorrect response during regular trials would lead to the return of the response cost to the participant, and hence prevent them from losing virtual Dollars overall. In addition, participants were informed that during devaluation blocks, there would be trials (consumption trials) where they could freely choose between two presented casino chips to exchange for money. They were also reminded that they should keep in mind which type of chip would not be exchanged for money in the respective block when choosing a chip. This instruction was followed by two exemplary consumption trials which constituted the end of the instructions.

The test phase consisted of four blocks. In each block, participants completed 48 regular trials (six repetitions of each bandit, order fully randomized within each block). Again, the task started with an amount of 10 virtual Dollars in the participant’s wallet. Responses were given using response devices in each hand. Each of the four blocks contained a devaluation block at the end in which one of the two outcomes (house or face) was selectively devalued.

Devaluation blocks were announced by a short text telling the participants that counterfeit chips were circulating, that the casino would not exchange houses or faces for money in this block, and that participants should hence avoid receiving the devalued outcome. Devaluation trials followed the same structure as regular trials with the following exceptions: In devaluation trials, an alarm bell was shown above the bandit throughout the full duration of each trial and above the fixation cross during inter-trial intervals. In devaluation trials, the amount of virtual money currently gained, the numerical representation of the (subtracted or returned) response cost, and the coin representing the returned response cost were not shown to ensure that devaluation trial choices were made in extinction. Instead, ‘???’ was displayed below the participant’s wallet and during the reward delivery phase if a casino chip was received (see Fig. 2b for an illustration of devaluation trials). All gained rewards were still added to the participant’s total sum which was displayed again after the devaluation block was completed.

In addition, each devaluation block contained two consumption trials as described above. These trials were added to test participants’ understanding of the devaluation instructions: In devaluation blocks where houses were devalued, they should always choose the face chip and vice versa in order to obtain a reward. Upon choosing the non-devalued option, participants received a high (100) reward in one of the two trials and a low (10) reward in the other trial. Again, this reward was not shown but still added to the participant’s total sum which was displayed again after the devaluation block.

Each devaluation block consisted of eight trials (one presentation of each of the eight stimuli, hence four trials with devalued outcomes and four trials with still valuable outcomes). Across devaluation blocks, houses and faces were devalued in alternating fashion. Whether houses or faces were devalued in the first block was pseudorandomized across participants.

Each experimental block of the test phase lasted around 15 min. At the end of the test phase, participants were informed about their response accuracy and the additional payout gained during the test phase (calculated as described above). Including the collection of localizers, field maps, and an anatomical scan, the test phase in the scanner lasted around 80 to 90 min.

### 2.4 fMRI data collection

We collected fMRI data during the test phase of the experiment using a 3T Siemens Prisma scanner with a 32-channel head coil and a gradient echo planar sequence with the following parameters: TE = 25  ms, TR = 2.07  s, flip angle $=\;80^{\circ }$, voxel size = 3 × 3 × 3.2  mm corresponding to a slice thickness of 3.2 mm (gap: 0.64 mm), matrix size = 64 × 64 . Each volume contained 36 slices measured in descending order. The number of collected volumes per run varied across participants depending on the duration of the self-paced breaks between the experimental blocks (roughly between 920 and 1050 volumes per run).

We also collected anatomical T1-weighted images using a sequence with the following parameters: TE = 2.26  ms, TR = 1.9  s, flip angle $=\;9^{\circ }$, voxel size = 1  mm isotropic, and FoV = 224 × 256 . Additionally, field maps with identical resolution to functional images were acquired at the beginning of each of both experimental runs (TR = 439  ms, short TE = 5.32  ms, long TE = 7.78  ms, and flip angle = $45^{\circ }$).

Since the scanning phase of the experiment consisted of four blocks, the fMRI scan was divided into two runs containing two blocks each. A T1-weighted anatomical scan was collected at the end of the experimental procedure. Fieldmaps were collected at the beginning of each run.

### 2.5 Behavioral data analysis

For each of the 59 included participants, we computed the following behavioral indices of task performance: 1) overall task performance in the training phase (standard blocks and reward choice trials), and 2) overall task performance in the test phase. In the sample of 57 participants included in the analysis of devaluation blocks, we further computed: 1) the percentage of ’slips-of-action’ (SOAs), hence trials, where participants pressed the initially learned button even though the outcome was devalued relative to trials with devalued outcome where a response was indicated (excluding missing trials), and 2) the percentage of error trials where participants pressed the initially incorrect button even though the outcome was not devalued relative to trials with still valuable outcome where a response was indicated (excluding missing trials). In addition, we computed response times for correct responses in trials with devalued and still valuable outcomes. For both error percentages and response times, we additionally computed a 2 (devalued vs. still valuable) × 2 (high vs. low reward) ANOVA to test for a potential motivational influence of reward magnitude on devaluation block performance. This analysis was conducted because an effect of value has been reported in previous research (Meier et al., 2022).

For each participant, we computed the mean accuracy of explicit knowledge of the correct response, outcome, and reward associated with each stimulus in both task questionnaires. Finally, we calculated the mean of each NASA-TLX subscale across participants to assess subjective task effort.

As our main dependent variable, we computed an index of behavioral goal-directedness as the number of trials where participants correctly adjusted their behavior to a devalued outcome relative to the total number of given responses in trials with devalued outcome. This index was z-standardized and used in all analyses linking neural signatures of outcome anticipation to behavioral goal-directedness.

### 2.6 fMRI data analysis

#### 2.6.1 Preprocessing

FMRI data were preprocessed using fMRIPrep (version 20.2.7, Esteban et al., 2019). We did not run Freesurfer’s surface reconstruction and used fieldmap-based distortion correction. All magnitude files used to compute fieldmaps were brain-extracted prior to preprocessing using FSL bet. We further used MRIQC (version 24.0.0, Esteban et al., 2017) to conduct visual quality checks of MRI data. We had initially planned to exclude participants for excessive head motion (average framewise displacement >0.5  mm); however, no participant reached this criterion. Instead, one participant was excluded for excessive motion peaks (FD >1.5  cm in both fMRI runs).

Following these preprocessing steps, we used a Nipype (Gorgolewski et al., 2011) implementation of SPM 12 functions to estimate a first-level denoising GLM for each participant. This GLM included the following nuisance regressors: translation and rotation as well as their respective first-order derivatives in the x, y, and z dimensions, time series extracted from CSF and white matter, and a global signal regressor as well as a constant term for each of the two scanning runs. Residual images from this denoising analysis were used as input for all multivariate analyses described below. For the univariate analysis, preprocessed data were smoothed using a 6 mm Gaussian kernel before estimating the GLM described below. In both cases, fMRI data were high-pass filtered at 1/​128 Hz. Multivariate classification analyses were conducted using the decoding toolbox for SPM12 (TDT, version 3.999F, Hebart et al., 2015).

#### 2.6.2 Anatomical ROIs

In addition to whole-brain analyses, multivariate analyses were also computed for the following anatomical ROIs of the goal-directed system: right posterior LPFC (10 mm sphere from McNamee et al., 2015; note that the original authors labeled this area dlPFC) and contralateral sphere in the left hemisphere, vmPFC (10 mm sphere from McNamee et al., 2015), left and right anterior caudate nucleus, and left and right angular gyrus (anatomical ROI created from the AAL atlas using SPM WFUPickAtlas; anterior: y > 2, posterior: y ≤ 2, de Wit et al., 2012). Next to these main confirmatory ROIs, the left and right posterior caudate were investigated in an exploratory analysis (anatomical ROIs created from the AAL atlas using SPM WFUPickAtlas; anterior-posterior distinction as reported above). The angular gyrus was chosen in addition to regions identified by McNamee et al. (2015) because it had previously been implicated in outcome-based decision-making (e.g., Zwosta et al., 2015, 2018). To account for a slight mismatch between the SPM and fMRIPrep anatomical templates, transformation parameters were computed by registering the SPM anatomical template to the fMRIPrep template using ANTs. The resulting transformation parameters were then applied to the anatomical ROI masks.

#### 2.6.3 Univariate analyses: Neural activation associated with successful behavioral adjustment to outcome devaluation

Using SPM12, we computed a GLM containing regressors corresponding to the following experimental conditions: 1) stimulus onset of correct trials in regular blocks; 2) stimulus onsets of trials with correct behavioral adjustment to a devalued outcome; 3) stimulus onsets of trials representing an SOA; 4) stimulus onset of trials with correct answer to a still valuable outcome during devaluation blocks; 5) stimulus onset of trials with an erroneous response to still valuable outcomes during devaluation blocks; and 6) incorrect and missing trials from regular blocks as well as missing responses during devaluation blocks. All onsets were modeled as a stick function with duration zero. In addition, the model included nuisance regressors for translation and rotation in x, y, and z direction, time series from CSF and white matter, a global signal regressor, as well as a constant term for each experimental run, and data were high-passed filtered at 1/​128 Hz. For each participant, we estimated the following contrasts of interest: correct adjustment to devalued outcome > baseline, correct adjustment to devalued outcome > correct response to still valuable outcome, and correct adjustment to devalued outcome > SOA. The last contrast was also computed in reverse. Subsequently, we computed group-level one-sample t-tests (results thresholded at p<.001 uncorrected and FWE-corrected at the cluster-level). Note that contrasts were only computed for experimental runs in which all regressors used for contrast estimation were existent (i.e., if one participant did not make mistakes in devalued trials in run two, the third contrast was only estimated from the regressors corresponding to run one in this participant).

#### 2.6.4 Multivariate analyses: Neural representations of anticipated outcomes

##### 2.6.4.1 Manipulation check

To test for regions significantly encoding outcome stimuli (houses vs. faces), we identified for each participant residual images from the denoising GLM corresponding to TRs two and three after outcome presentation in each trial roughly capturing the peak of the expected hemodynamic response function in response to outcome image presentation (each TR = 2.07 s, hence a time window spanning 4.14 s with a temporal distance of 2.07 s from the image during which the outcome onset occurred). We averaged these two images and subjected the resulting image to an MVPA analysis conducted separately for each participant. Here, we used 4-fold leave-one-block-out cross-validation and employed a kernel classifier to test for regions differentiating between house and face outcomes. We used the default kernel classifier employed by TDT which is a linear support vector machine implemented by LIBSVM; for more details on the default settings of TDT, please refer to Hebart et al. (2015) and the software documentation. This classification analysis was conducted as a whole-brain searchlight analysis using an 8 mm searchlight radius. The resulting accuracy maps were subsequently smoothed using a 6 mm Gaussian FWHM kernel and subjected to a second-level one-sample t-test (results thresholded at p<.001 uncorrected and FWE-corrected at the cluster-level) using SPM12.

##### 2.6.4.2 Identifying anticipatory outcome representations

**Fig. 3.**
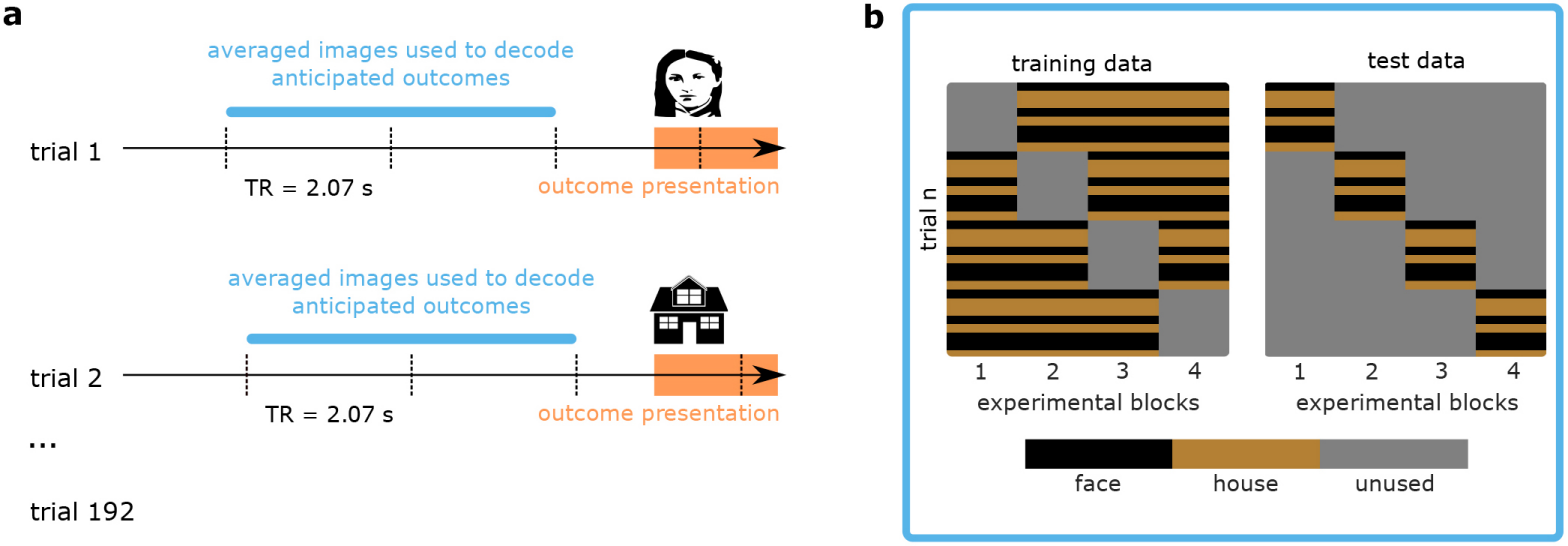
Schematic depiction of the MVPA procedure. (a) Data preparation for MVPA. We identified the two residual images corresponding to the two TRs preceding the TR during which the outcome onsets were presented in each trial. These images were averaged and used as input to the MVPA analysis (dashed lines represent TRs); (b) Schematic depiction of the cross-validation procedure. The averaged residual images were subjected to an MVPA using a 4-fold leave-one-block-out cross-validation procedure. Note that trials in each block were presented in fully randomized order not blocked; panel b does not depict trials in their actual order of presentation in each block.

We then averaged the two residual images corresponding to the two TRs preceding outcome presentation (hence spanning a time window of 4.14 s preceding the TR during which the outcome onset occurred) to approximate sustained neural activation corresponding to the combined outcome anticipation phase and response window in each trial (see Fig. 3a). The averaged images were then subjected to an MVPA analysis that was conducted separately for each participant. Again, we used 4-fold leave-one-block-out cross-validation (see Fig. 3b) and employed a kernel classifier to test for regions differentiating between anticipated house and face outcomes during this anticipatory phase. Here, we used the same classifier as described in Section 2.6.4.1.

This classification analysis was conducted as a whole-brain searchlight using an 8 mm searchlight radius, as confirmatory ROI analysis in bilateral posterior LPFC, vmPFC, bilateral anterior caudate, and angular gyrus, and additionally as exploratory ROI analysis in bilateral posterior caudate. Searchlight output maps were smoothed using a 6 mm Gaussian FWHM kernel and subjected to an SPM second-level one-sample t-test (results thresholded at p<.001 uncorrected and FWE-corrected at the cluster-level). Classification results from ROI analyses were tested for statistical significance using a one-sample Wilcoxon sign-rank test. The resulting p-values were Bonferroni-corrected for seven comparisons.

To test for associations with devaluation performance, we used the computed index of goal-directed behavior as a covariate of interest in an SPM second-level model and tested this covariate for statistical significance in a whole-brain analysis. Here, we further applied small volume correction for the regions identified in the previous analysis. That is because we expected an association between goal-directed behavior and neural activation reflecting outcome anticipation specifically in regions where outcome anticipation reached statistical significance in the first place.

For a priori ROI analyses, we computed Spearman correlations between participants’ individual classification accuracy and the respective behavioral index of goal-directedness in each ROI. Again, results from ROI analyses were Bonferroni-corrected for seven comparisons.

Finally, we separately computed the whole-brain decoding analysis described above for trials with high and low reward magnitude. We then computed a second-level paired t-test to test for brain regions where anticipatory outcome encoding differed between the high and low reward conditions.

##### 2.6.4.3 Control analysis

By design, responses, outcomes, and rewards were orthogonalized in this study. Hence, classification accuracy reflecting anticipatory outcome encoding could not reflect differential responses or reward magnitudes associated with the outcomes across trials. However, the house outcomes were associated with four geometric shape stimuli and the face stimuli were associated with the other four geometric shape stimuli. That is, neural activation in the identified regions might have differentiated between the four stimuli associated with a house and the four stimuli associated with a face outcome instead of with the anticipated outcome identity itself. To assess this potential confound, we computed decoding analyses differentiating between two groups of four trial sequences each. These groups were formed such that both groups only differed in stimulus information: The trial sequences in each group contained exactly two occurrences of a left or right response, house or face outcome, and high or low reward magnitude. Hence, all contingency features except for stimulus identity occurred equally often in both groups. Consequently, significant decoding accuracy between both groups should reflect stimulus identity and none of the other contingency features since they were represented equally across both groups. As there were eight possible respective groupings, eight separate analyses were computed.

For these analyses, we used the same classifier and cross-validation approach as reported above. Decoding accuracy across all eight analyses was averaged for each participant. The averaged accuracy maps were smoothed using a 6 mm Gaussian kernel and subjected to a second-level one-sample t-test (results thresholded at p<.001 uncorrected and FWE-corrected at the cluster-level). Hence, significant clusters resulting from this analysis represented brain areas, where decoding accuracy for anticipated outcomes as identified by the previous main analysis could potentially be confounded by stimulus identity (or full contingency sequences).

##### 2.6.4.4 Time-span analysis

In addition to the analyses reported above, we conducted a so-called ‘time-span’ decoding analysis (McNamee et al., 2015). Here, we used the two images after outcome presentation (corresponding to the data used for the manipulation check) as the training dataset and the anticipatory time window before outcome presentation (i.e., the anticipatory window used for the analyses identifying anticipatory neural outcome representations) as the test dataset. Again, the same classifier as described above was used and 4-fold leave-one-block-out cross-validation was employed. The rationale of this analysis was to assess whether we could identify brain areas, where anticipatory neural encoding patterns reflected neural patterns elicited by the actual visual presentation of the outcome. Here, we were specifically interested in perceptual areas. For example, it has been shown that house or face anticipation results in elevated activation in corresponding perceptual areas (see Waszak et al., 2012, for review), or that odor expectations are encoded in the piriform cortex (Howard & Kahnt, 2017). Hence, we conducted this analysis as a whole-brain analysis and applied additional small volume correction for anatomical ROI masks representing the left and right fusiform gyrus.

## 3 Results

### 3.1 Explicit contingency knowledge

After the initial training phase, participants’ mean accuracy for explicitly indicating task contingencies associated with each of the eight stimuli was 98.94% (SD=3.51% ) for responses (left or right key), 88.98% (SD=21.41% ) for outcomes (houses or faces), and 96.40% (SD=10.13% ) for rewards (high or low reward). Note that five participants only performed at chance or below for the associated outcomes. However, we did not exclude these participants from the analyses since they correctly indicated task contingencies at the end of the experiment. At the end of the experiment, overall accuracy was 99.36% (SD=3.61% ) for responses, 98.73% (SD=5.03% ) for outcomes, and 97.67% (SD=8.83% ) for reward magnitude. Thus, overall, participants were able to learn task contingencies with sufficient accuracy throughout the training and scanning phase of the experiment.

### 3.2 Task performance

#### 3.2.1 Training phase

Response accuracy in the training phase was high overall (M=92.57% , SD=7.77% ). In reward choice trials, participants succeeded at choosing the bandit associated with the higher reward magnitude with an accuracy of 92.06% (SD=10.16% ).

#### 3.2.2 Test phase

Overall, task performance in regular blocks during the scanning phase was very high (M=98.46% , SD=2.32% ) indicating participants’ sustained attention throughout the full experiment. Task performance did not significantly differ between trials with high and low reward, t(58) = 0.59, p = .56.

#### 3.2.3 Devaluation blocks

**Fig. 4.**
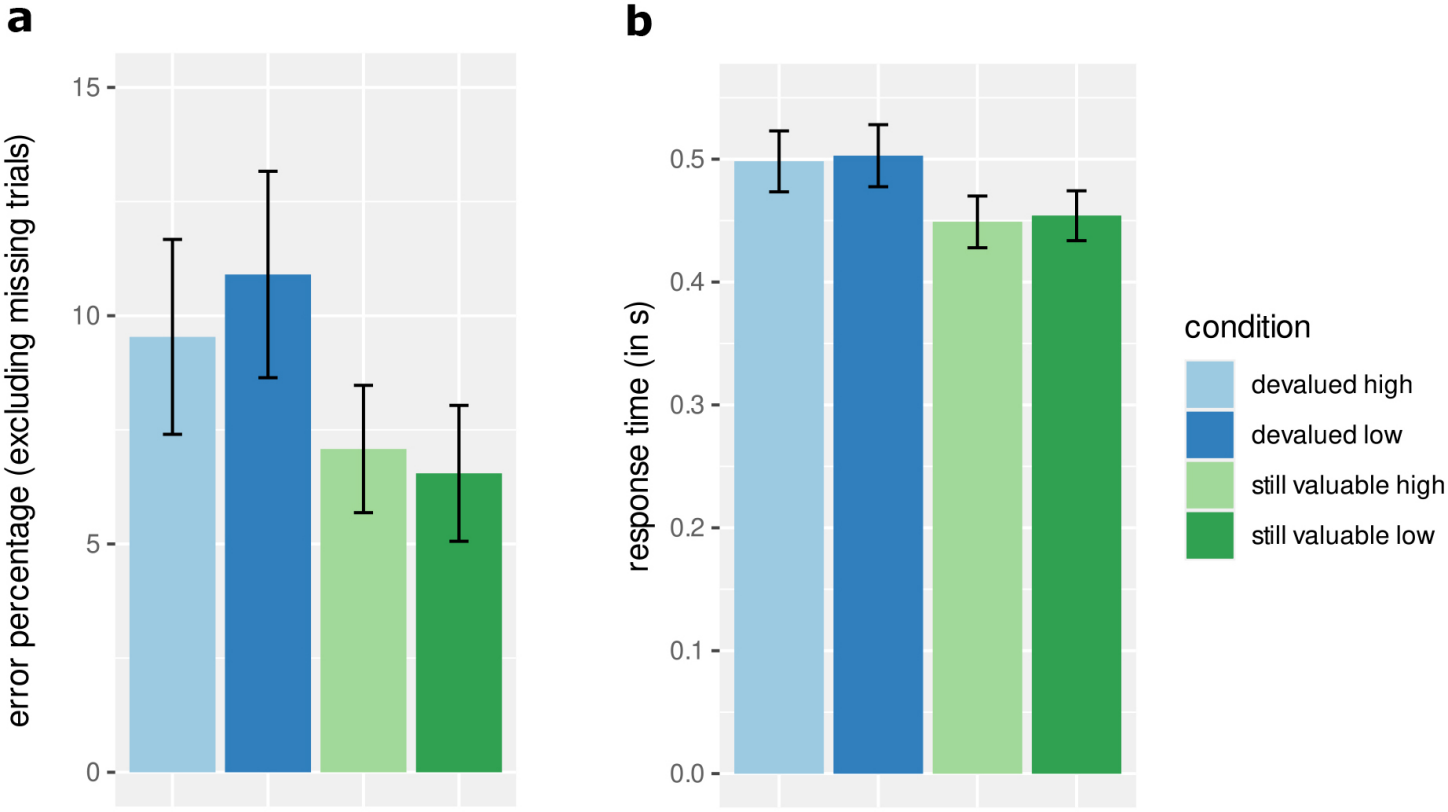
(a) Mean error percentage in devaluation blocks according to value and reward magnitude; (b) Mean response time in correct trials according to value and reward magnitude. Conditions: devalued = trials with devalued outcome, still valuable = trials in devaluation blocks with still valuable outcome, high = associated with high reward magnitude during training, low = associated with low reward magnitude during training. Error bars represent the standard error of the mean.

In trials with a devalued outcome, participants’ percentage of SOAs was relatively low overall (M=10.17% , SD=14.73% , range: 0 to 63.64%) indicating that participants were mostly successful at adjusting their response behavior to changes in outcome value. This result further suggests that the devaluation procedure was, indeed, effective. In trials with still valuable outcomes in devaluation blocks, overall error percentage was 6.79% (SD=9.76% , range: 0 to 50%). Adding reward magnitude as an additional factor, a two-way repeated-measures ANOVA confirmed a significant main effect of outcome devaluation, F(1, 56)=4.54 , p=.04 , $\eta_{p}^{2}=.08$ , indicating that participants had a higher percentage of SOAs in trials with devalued compared to errors in trials with a still valuable outcome. There was no significant main effect of reward magnitude or interaction between devaluation and reward magnitude, all p>.2 (see Fig. 4a for a visualization of the resulting pattern for error percentages, and Supplementary Figs. S2a and S3a for distribution plots).

For response times in correct trials during devaluation blocks, we observed a significant main effect of devaluation, F(1, 56)=10.70 , p=.002 , $\eta_{p}^{2}=.16$ , indicating slower response times when a correct answer in a devalued trial (M=498.01 ms, SD=167.75 ms) compared to a still valuable trial (M=452.41 ms, SD=144.20 ms) was given. This finding indicates that correctly adjusting behavior to a devalued outcome might require suppressing a habitual response, slowing overall response times down. Again, there was no main effect of reward magnitude or interaction between both factors, all p>.6 (see Fig. 4b for a visualization of the resulting pattern for response times, and Supplementary Figs. S2b and S3b for distribution plots).

In consumption trials, overall accuracy was very high (M=98.68% , SD=4.53% ). These results indicate that participants correctly remembered the devalued outcome and that the devaluation manipulation successfully induced slips-of-action in trials with devalued outcomes. However, participants still acted very goal-directed as shown by the relatively high accuracy in adapting their response behavior to changes in outcome value.

Finally, our main index of goal-directed behavior indicated highly goal-directed behavioral control on average (M=89.83% , SD=14.73% ). Supplementary Figure S4 shows a distribution of the number of correct adjustments to devalued outcomes, SOAs, missing responses, and the index of behavioral goal-directedness per experimental block.

### 3.3 Subjective effort ratings

On the NASA-TLX scales ranging from one to 20, participants rated the mental demand of the task as medium (M=10.05 , SD=3.91 ). Participants also reported relatively high satisfaction with their task performance (M=16.47 , SD=2.99 ) as well as medium overall effort (M=9.36 , SD=4.27 ) and low task frustration (M=4.42 , SD=3.44 ).

### 3.4 Neural activation associated with successful behavioral adjustment to outcome devaluation

In the GLM estimating neural activation at stimulus onset in each trial, the contrast correct adjustment to devalued outcome > baseline yielded widespread significant activation clusters across the whole brain, including areas typically considered part of the fronto-parietal network (regions in the dorsolateral prefrontal cortex) and areas related to conflict processing (insula and supplementary motor area). Supplementary Table S1 shows all significant activation peaks. In addition, two statistically significant clusters yielded greater activation in trials where behavior was correctly adjusted to devalued outcomes compared to trials where a correct answer for a still valuable outcome was given (Calcarine, peak: x = 0, y = -96, z = 4, p=.01 , cluster size: 168 voxels; Cerebellum R, peak: x = 14, y = -58, z = -22, p=.01 , cluster size: 164 voxels). No brain regions showed differential activation for correct adjustment to devalued outcomes versus SOA trials. Note, however, that the latter two contrasts relied on a very small number of trials and that our experimental design was hence not optimized to analyze neural activation during devaluation block trials.

### 3.5 Manipulation check: Outcome representations

A whole-brain analysis testing for regions where house and face outcomes could be decoded with statistically significant accuracy after outcome presentation resulted in a significant cluster comprising bilateral occipital cortices extending into bilateral fusiform gyri as expected (peak: x = 40, y = -74, z = -6, p<.001 , cluster size: 17628 voxels) demonstrating that the stimuli we used were significantly encoded visually and in occipitotemporal regions known to be involved in (object) category representation (Ishai et al., 2000).

### 3.6 Anticipatory outcome representations

#### 3.6.1 Whole-brain classifier

**Fig. 5.**
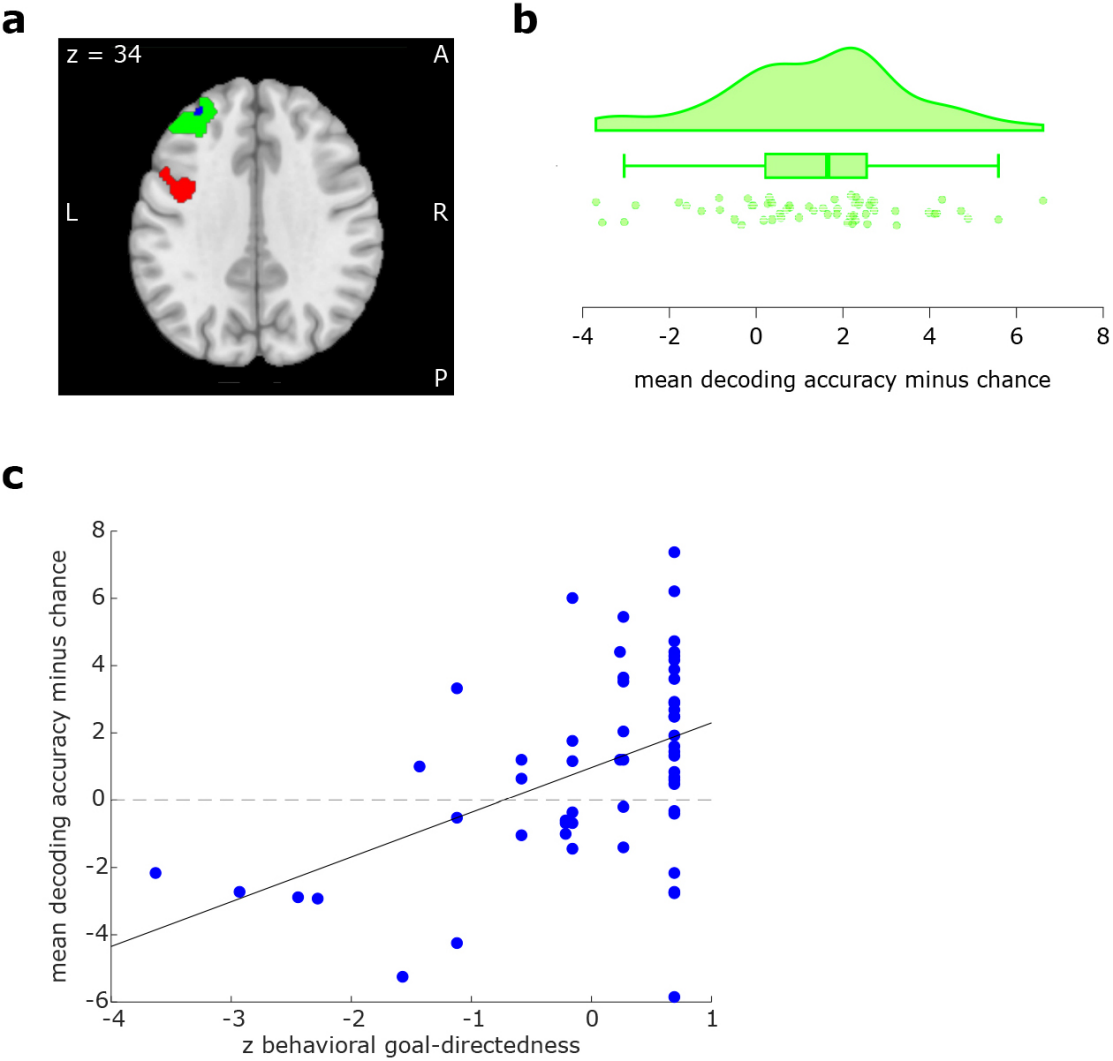
(a) Anticipatory outcome encoding. Green voxels = left dlPFC cluster where anticipatory decoding accuracy was statistically significant in a whole-brain analysis; blue voxels: subset of voxels where decoding accuracy was predictive of behavioral goal-directedness when applying small volume correction for the green cluster; red voxels = significant posterior LPFC cluster resulting from the control analysis (see Section 3.6.4); (b) Distribution of mean decoding accuracy minus chance in percent. Values extracted from voxels constituting the cluster depicted in green (in the full sample, N = 59); (c) Association between goal-directed performance and mean decoding accuracy minus chance. Valued extracted from the voxels depicted in blue in panel a; dashed lines represent chance level (50%), solid lines represent the line of best linear fit; the brain-behavior correlation depicted in panel c was computed in a sample of 57 participants after the exclusion of two participants who failed to understand the devaluation instruction.

Using a whole-brain searchlight classifier, we could identify three clusters, where response outcomes were significantly encoded before actual outcome presentation: right lingual gyrus (peak: x = 24, y = -82, z = -4, p<.001 , cluster size: 3832 voxels), left lingual gyrus (peak: x = -14, y = -86, z = -6, p<.001 , cluster size: 5234 voxels), and left dlPFC (peak: x = -40, y = 36, z = 32, p=.04 , cluster size: 361 voxels); see Figure 5a for a visualization of the dlPFC cluster (green voxels) and Fig. 5b for a visualization of the distribution of decoding accuracy (minus chance) in this cluster.

**Fig. 6.**
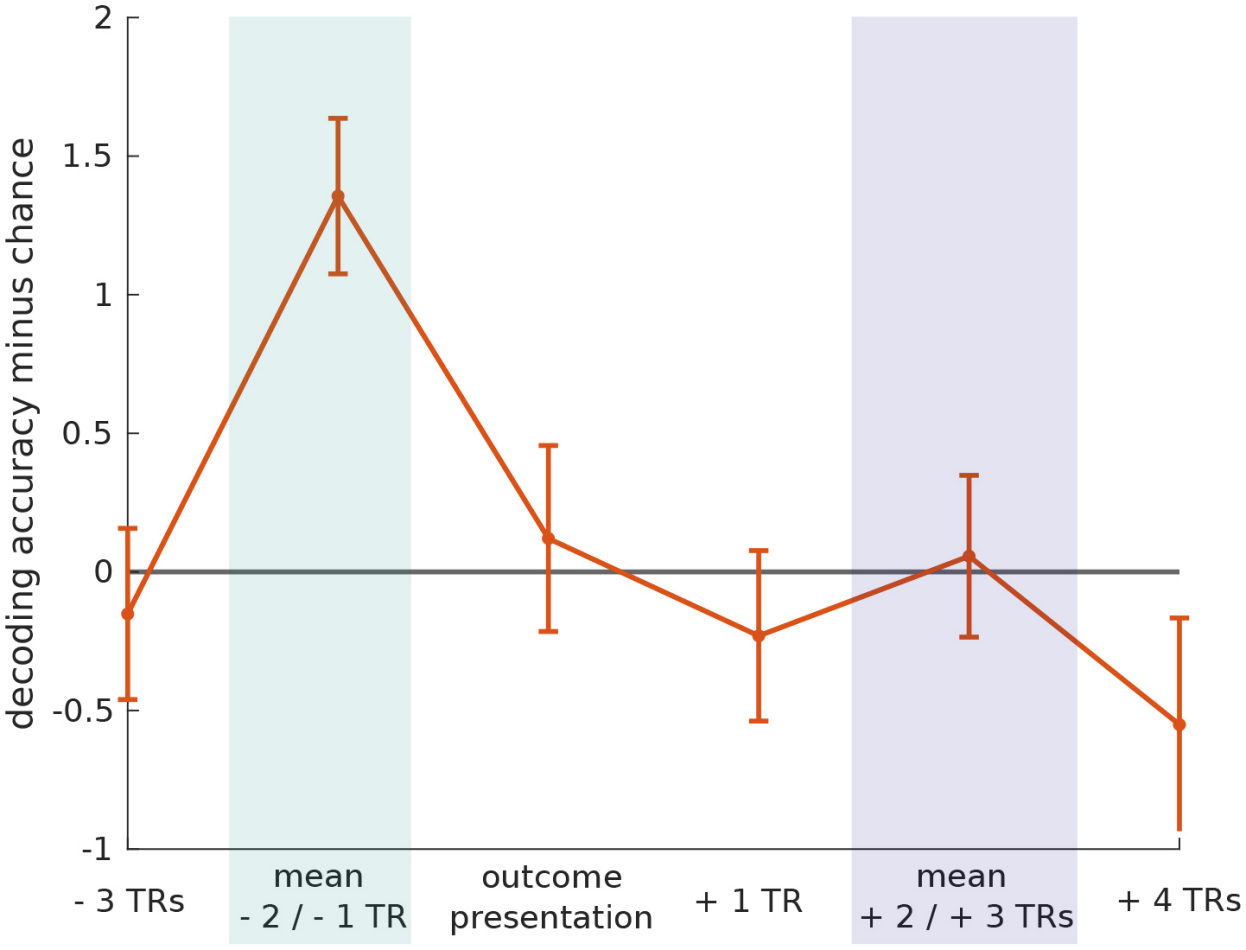
Time course of mean dlPFC decoding accuracy minus chance across participants and time. Error bars indicate the standard error of the mean. The blue bar indicates the outcome anticipation phase used for the main decoding analyses, and the purple bar indicates the time window after outcome presentation used for the manipulation check.

Figure 6 shows the time course of mean decoding accuracy of the full dlPFC cluster. Decoding accuracy is shown for time points at the beginning of the trial (three TRs before outcome presentation), during outcome anticipation (mean of the two images corresponding to the two TRs preceding outcome presentation), during and one TR after outcome presentation, during the outcome representation phase used for the manipulation check, and four TRs after outcome presentation. As recognizable in Figure 6, dlPFC encoding of action outcomes was specific to the anticipatory phase of the trial timeline.

Adding the index of behavioral goal-directedness as a covariate to test for an association of neural encoding strength and devaluation performance did not yield any significant cluster at the level of the whole brain. In addition, we did not find any regions where anticipatory outcome decoding accuracy significantly differed between trials with high versus low reward magnitude.

#### 3.6.2 Follow-up analysis: Small volume correction

Applying small volume correction for the two previously identified clusters in the lingual gyrus did not yield any statistically significant regions where voxel-wise decoding accuracy was significantly associated with behavioral goal-directedness. However, applying small volume correction for the dlPFC cluster identified during the previous analysis yielded two statistically significant clusters (see Fig. 5a blue voxels) where higher decoding accuracy was significantly associated with higher behavioral goal-directedness (peaks: x = -32, y = 46, z = 24, p=.02 and x = -36, y = 46, z = 36, p=.03 , small volume corrected, cluster sizes 12 voxels and one voxel, respectively, uncorrected for testing multiple ROIs and hence to be treated as preliminary and with caution); see Figure 5b for a visualization of the association between mean decoding accuracy and behavioral goal-directedness in this cluster. Hence, in this region, putatively, decoding accuracy as a neural marker of outcome anticipation significantly predicted behavioral goal-directedness.

#### 3.6.3 A priori ROI analyses

Conducting a priori ROI analyses, we found significant anticipatory outcome encoding in the left posterior LPFC ROI (p=.02 , Bonferroni-corrected for seven comparisons) hence contralateral to the right ROI described by McNamee et al. (2015). It is important to note that this a priori ROI is non-overlapping with and significantly posterior to the left dlPFC region we identified in the whole-brain analysis described above. In addition, decoding accuracy in the right angular gyrus reached statistical significance (p<.01 , Bonferroni-corrected for seven comparisons). Note that decoding accuracy in the left angular gyrus reached statistical significance before correcting for multiple testing (p=.02 , uncorrected). Computing rank correlations between decoding accuracy and behavioral goal-directedness did not yield any statistically significant association for any of our ROIs of interest (all uncorrected p-values >  .2).

#### 3.6.4 Control analysis

To rule out a potential confound resulting from stimulus identity encoding, we conducted a whole-brain control analysis revealing clusters where anticipatory outcome encoding identified in the previous analyses might theoretically have been driven by differences in stimulus identity (geometric shape stimuli) alone. This analysis yielded statistically significant clusters in the bilateral visual cortices (peak: x = -20, y = -90, z = -6, p<.001 , cluster size: 22037 voxels) and in a left posterior LPFC region (peak: x = -54, y = 14, z = 24, p<.01 , cluster size: 633 voxels, see Fig. 5a red voxels). Applying small volume correction for the ROIs reported as significant in the ROI analysis yielded a statistically significant cluster in the right and left angular gyrus (peak right: x = 30, y = -56, z = 48, p<.01 , cluster size: 365 voxels; peak left: x = -32, y = -60, z = 44, p<.01 , cluster size; 191 voxels, both small volume corrected) and the left posterior LPFC ROI derived from McNamee et al. (2015), peak: x = -44, y = 2, z = 32, p<.01 , cluster size: 226 voxels, small volume corrected. This result suggests that the significant results in visual cortices, right angular gyrus, and the a priori left posterior LPFC ROI might have been driven by stimulus instead of outcome identity. Notably, no voxels in the left dlPFC ROI we identified in our whole-brain classification analysis survived statistical thresholding in the control analysis indicating that this result was genuinely driven by outcome anticipation. To double-check this claim, we conducted exploratory Bayesian one-sample t-tests in JASP (version 0.19.1.0). Here, we extracted mean decoding accuracy from the control analysis and from the main analysis testing for anticipatory outcome encoding in the dlPFC cluster identified during the main analysis. For mean activation from the control analysis, we examined evidence in favor of the null hypothesis that stimulus identity did not drive neural activation patterns in this region (decoding accuracy equal to zero) over the alternative hypothesis that decoding accuracy for this region was significantly greater than zero. For the main analysis, we examined evidence in favor of the alternative hypothesis that mean decoding accuracy in this region for anticipated outcomes was greater than zero over the null hypothesis that decoding accuracy in this region was equal to zero. In line with our expectations, this analysis yielded strong evidence (BF = 10.31) in favor of the null hypothesis for the control analysis and decisive evidence (BF = 3577.79) in favor of the alternative hypothesis for the main analysis. This result clearly indicates that we identified a region in left dlPFC where action outcomes were encoded during outcome anticipation independent of stimulus identity.

#### 3.6.5 Time span decoding analysis

We did not find any whole-brain statistically significant cluster when testing a classifier trained after outcome presentation on data corresponding to the outcome anticipation phase. Small volume correction did not reveal any statistically significant results in any of the a priori ROIs, in the fusiform gyrus, or the left dlPFC region identified in the main analysis.

## 4 Discussion

In this study, we showed that: 1) Anticipated response outcomes were neurally represented in a region of the goal-directed system in the brain, namely the left dorsolateral prefrontal cortex; 2) Anticipatory neural outcome encoding in this region significantly predicted goal-directed behavior during selective outcome devaluation.

We identified a region in left dlPFC where action outcomes were significantly encoded in an anticipatory manner. It is noteworthy that this region was different from our (more posterior) a priori posterior LPFC ROI where neural response patterns during outcome anticipation were most likely driven by stimulus identity or the entire S-R-O-Reward contingency. The dlPFC is part of a multiple-demand network in the brain and has been shown to encode important aspects of any given task such as task identity and stimulus features (e.g., Shashidhara et al., 2024) or task rules (Mian et al., 2014) to name a few examples. More generally, computational models of the dlPFC have considered this region as important in encoding task sets or representations that drive goal-directed action selection (Botvinick & An, 2008; Miller & Cohen, 2001; Morris et al., 2014; Soltani & Koechlin, 2022) presumably via its connectivity with motor areas (Miller & Cohen, 2001; Morris et al., 2014). This assumption fits well with our finding that anticipatory outcome encoding in this region positively predicted participants’ goal-directed behavioral control. Previous studies have already demonstrated anticipatory outcome encoding in dlPFC (e.g., McNamee et al., 2015; note, however, that we labeled this region posterior LPFC here), linked dlPFC activation strength to the expression of goal-directed behavior (Watson et al., 2018), and have demonstrated that anticipatory reward representations in dlPFC were significantly associated with subsequent value-guided choices (Kahnt et al., 2011). Further, the same left dlPFC region identified here has been linked to working-memory dependent impairments in model-based behavior when disrupted using TMS (Smittenaar et al., 2013) supporting the causal role of this region in enabling goal-directed behavioral control. In addition, a left dlPFC region in close proximity but slightly posterior to the cluster identified here has been linked to target identity anticipation (in this case color distributions) in a visual search task (Witkowski & Geng, 2023) further backing the role of this general brain area in outcome identity anticipation. However, no other study has, to the best of our knowledge, linked outcome anticipation in dlPFC to successful behavioral adjustment to selective outcome devaluation so far.

In our study, the significant association between anticipatory outcome encoding and goal-directed behavior was limited to a small set of voxels in left dlPFC. In a more posterior cluster neural activation patterns were presumably driven by stimulus identity instead and were unrelated to goal-directed behavior. The (lateral) prefrontal cortex is a heterogeneous structure and a rostro-caudal processing gradient has been suggested with higher levels of abstraction or episodic control in more rostral compared to more caudal areas; see Badre and Nee (2018) or Koechlin et al. (2003) for a brief overview. It is tempting to argue that this might be the reason why we identified (abstract) anticipatory activation in a more anterior dlPFC region while more posterior activation was primarily driven by stimulus identity. On the other hand, stimulus identity in our study signaled the full contingency of any given trial in our task, such that brain regions identified in our control analysis might also have encoded the full S-R-O-Reward contingency task set in each trial. In any case, it is a given that distinct areas of the lateral prefrontal cortex encode different aspects of a given task set or contingency structure; see also Soltani and Koechlin (2022). In this study, we focused on outcome identity anticipation, but future research could employ similar experimental setups to disentangle the encoding of different types of associative content across distinct areas of the lateral prefrontal cortex with respect to predicting goal-directed behavior in outcome devaluation paradigms. Nevertheless, our findings stress the role of the dlPFC in the successful implementation of goal-directed behavior via anticipatory outcome encoding. This is an important finding because there is less agreement about the role of dlPFC in goal-directed behavior compared to more extensively studied regions in the field, such as the vmPFC (Griffiths et al., 2014).

Contrary to our expectations, we did not observe evidence for significant anticipatory outcome encoding or an association of encoding strength and goal-directed behavior in any of our a priori ROIs or elsewhere in the brain. Especially in the vmPFC, a lack of significant results is surprising given the well-established role of this region in goal-directed behavioral control (de Wit et al., 2009, 2012; Howard & Kahnt, 2017; Liljeholm et al., 2011; Morris et al., 2014; Tanaka et al., 2008; Zwosta et al., 2018), see O’Doherty (2011) for a brief review, especially its previously identified role in outcome anticipation (McNamee et al., 2015). In addition, recent theoretical accounts of the vmPFC have argued that this region serves the encoding of a cognitive map representing state spaces of the current task (Moneta et al., 2023; Schuck et al., 2016) or learned action policies (Hayden & Niv, 2021). There are two possible reasons why we did not find significant anticipatory outcome encoding in vmPFC despite the significant amount of empirical and theoretical work supporting the role of vmPFC in outcome anticipation. First, Schuck et al. (2016) have argued that the main role of vmPFC lies in encoding latent, unobservable aspects of the task space. In our experiment, all elements of the task space were fully observable to the participants which might explain our lack of results in vmPFC. Second, while trials in the devaluation blocks required goal-directed control, correct responses during regular blocks could be given via simple S-R associations. It is, hence, possible that vmPFC recruitment in these trials was low, since vmPFC is particularly engaged in experimental conditions relying on goal-directed associations (de Wit et al., 2009). This line of reasoning could potentially also explain why McNamee et al. (2015) reported anticipatory outcome encoding in vmPFC while we did not find a respective effect: In our study, participants completed a relatively long training phase and showed high knowledge of all task contingencies before entering the scanner. It can, consequently, be argued that response behavior was already highly automatic in our participants at the beginning of the scanning phase. On the contrary, McNamee et al. (2015) did not employ a training phase and the authors used different experimental sessions across which task contingencies changed and had to be relearned. Thus, even though our task entailed more stimuli and hence a higher number of contingencies to remember, it is possible that the task used by McNamee et al. (2015) engaged vmPFC more strongly than our task during regular blocks since their participants were scanned while still learning different contingencies across sessions. However, this argument would warrant an explanation for why vmPFC- but not dlPFC-driven goal-directed processes would be affected by participants’ level of response automatization. Alternatively, outcome identity encoding in dlPFC and vmPFC might differ in a time-dependent manner. McNamee et al. (2015) reported anticipatory outcome encoding in dlPFC already at stimulus presentation, whereas outcome encoding in vmPFC only arose after an action was selected. In this study, we investigated an anticipatory time window after response selection, so a vmPFC contribution would have been expected regardless. However, investigating outcome encoding with more fine-grained temporal resolution along the trial timeline could potentially shed more light on the null results reported in vmPFC in this study.

On a different note, we cannot with a 100% certainty differentiate between (Pavlovian) stimulus- and (instrumental) response-driven outcome anticipation here. Even though outcome delivery in our task was contingent upon making a correct response, each stimulus was associated with only one outcome (or error upon making an incorrect response). In theory, participants could thus have started to anticipate the associated outcome already upon stimulus presentation before selecting a behavioral response. McNamee et al. (2015), for example, used two different outcomes per stimulus depending on which one of two possible responses was given and hence (at least somewhat) circumvented this issue. However, the behavioral data reported here suggest that participants successfully used R-O associations to adjust their behavior to changes in outcome value. Since anticipatory neural activation during regular trials predicted this type of goal-directed behavior, outcome anticipation was likely at least to some degree of instrumental nature. In addition, Meier et al. (2022) used a similar experimental setup containing only one outcome category per stimulus and reported results in line with our findings. However, it is important to acknowledge that goal-directed behavior presumably relies on the dynamic interplay of multiple associative contents; for example, S-O, R-O, and O-R associations (Balleine & Ostlund, 2007), and that the formation of each of these association patterns might have contributed to the main result reported here in dlPFC.

It is noteworthy that our time-span decoding analysis, where we tested a classifier that was trained after outcome presentation in the dataset corresponding to the outcome anticipation phase, did not yield any statistically significant result. This finding is in opposition to a previous study using a similar approach (McNamee et al., 2015) or to a study using a localizer task to train the respective classifier on neural responses to the visual stimuli used later on in the experiment (Meier et al., 2022). This null result indicates that the neural activation patterns elicited by the presentation of the outcome itself were not directly activated during outcome identity anticipation. Since it has been shown that the expectation of house or face stimuli activates category encoding areas specific to houses or faces (see Waszak et al. (2012) for review), it could have been expected that the anticipation of house of face images would elicit a similar neural pattern as observed after actual outcome presentation, specifically in the visual cortex or fusiform gyrus. In the reward anticipation literature, there is an ongoing debate about whether reward anticipation evokes neural activation similar to the activation elicited by the reward itself or rather activation reflecting the underlying associative structure (Wang & Hayden, 2017). While our study was not designed to address this question directly and with respect to outcome identity anticipation, we did not find any evidence for sensory-specific anticipatory processes in visual brain areas.

This study has two main limitations that need to be addressed. First, while we explicitly focused on goal-directed behavior and not habit induction in this study, the logic of selective devaluation paradigms implies that slips-of-actions in trials with devalued outcomes are the direct result of an established habit competing with the intended goal-directed action. However, the validity of devaluation insensitivity as an index of habitual behavior in experimental paradigms typically employed in human research has been questioned recently (Ceceli & Tricomi, 2018; De Houwer et al., 2018; Nebe et al., 2024; Watson & de Wit, 2018; Watson et al., 2022), and the assumption that behavioral overtraining leads to strong(er) habit induction has been challenged (Gera et al., 2023; Pool et al., 2022). While this does not take away from our conclusions, it should be noted that our results do not necessarily imply that outcome anticipation in dlPFC was negatively associated with habitual behavior. Second, due to timing constraints related to the use of fMRI, we had to limit the regular and devaluation blocks of the test phase to a reasonable number of trials. Hence, behavioral adjustment to outcome devaluation was measured based on a relatively small number of trials. However, it is common in devaluation paradigms to use or analyze a relatively low number of trials with devalued outcome (e.g., de Wit et al., 2007, 2012, 2018; Liljeholm et al., 2015; Sjoerds et al., 2016) and moderating variables such as acute stress have been shown to affect outcome devaluation sensitivity only during a small number of initial devaluation trials (Schwabe & Wolf, 2009, 2010).

In addition, our participants displayed highly goal-directed behavior overall. While this is not a limitation per se and high levels of goal-directed behavior are common in studies using outcome devaluation in humans (e.g., de Wit et al., 2018), investigating boundary conditions of the effect reported here or studying whether this effect also underlies pronounced failure to exert goal-directed behavior could represent a research goal for future studies. For example, it would be possible to combine our approach with experimental manipulations or study populations where extensive alterations in goal-directed behavior have been reported. Some examples are acute stress (Meier et al., 2022; Schwabe & Wolf, 2009), sleep deprivation (Chen et al., 2017), or psychiatric conditions (Gillan et al., 2011; Sjoerds et al., 2013). Since we did not find behavioral or neural differences between trials with high and low reward magnitude, it could further be interesting to assess whether reward magnitude significantly interacts with other moderating variables; for example, Meier et al. (2022) showed differences in goal-directed behavior between stressed and non-stressed participants only in trials where a previously high reward was devalued.

## 5 Conclusion

In conclusion, we could show that a region in the left dorsolateral prefrontal cortex significantly showed neural representations indicative of outcome anticipation. Furthermore, anticipatory outcome encoding in this region significantly predicted goal-directed behavioral control. This finding demonstrates that regions of the goal-directed brain system enable successful behavioral adjustment to outcome devaluation via neural outcome anticipation. Our findings can be extended in future studies investigating whether the mechanism reported here can also explain conditions under which goal-directed behavior is typically reduced (e.g., under acute stress or in specific psychiatric populations).

## Supplementary Material

Supplementary Material

## Data Availability

The following data are openly available in the GIN repository (DOI: https://doi.org/10.12751/g-node.fq1sl6): aggregated behavioral data, experimental log files corresponding to the test phase, averaged residual images used for manipulation check and main as well as control and time-span analyses, and scripts corresponding to main behavioral and whole-brain multivariate fMRI analyses.
