# Supplementary material for: Exploring the link between anticipatory outcome encoding in the brain and goal-directed behavior during outcome devaluation
Source: Imaging Neurosci (Camb). 2025 Oct 24;3:IMAG.a.956. doi: 10.1162/IMAG.a.956 (PMC12556683; doi:10.1162/IMAG.a.956)
Supplement: Supplementary Material [file IMAG.a.956_supp.pdf]

Supplementary Material

Stimulus Set

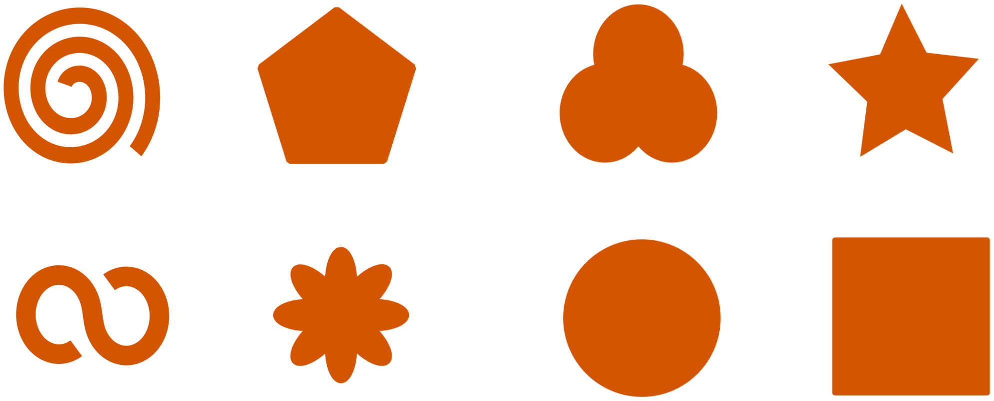

Figure S1: Depiction of all eight geometric shapes used as stimuli in this study.

Distribution of Error Percentage and RTs

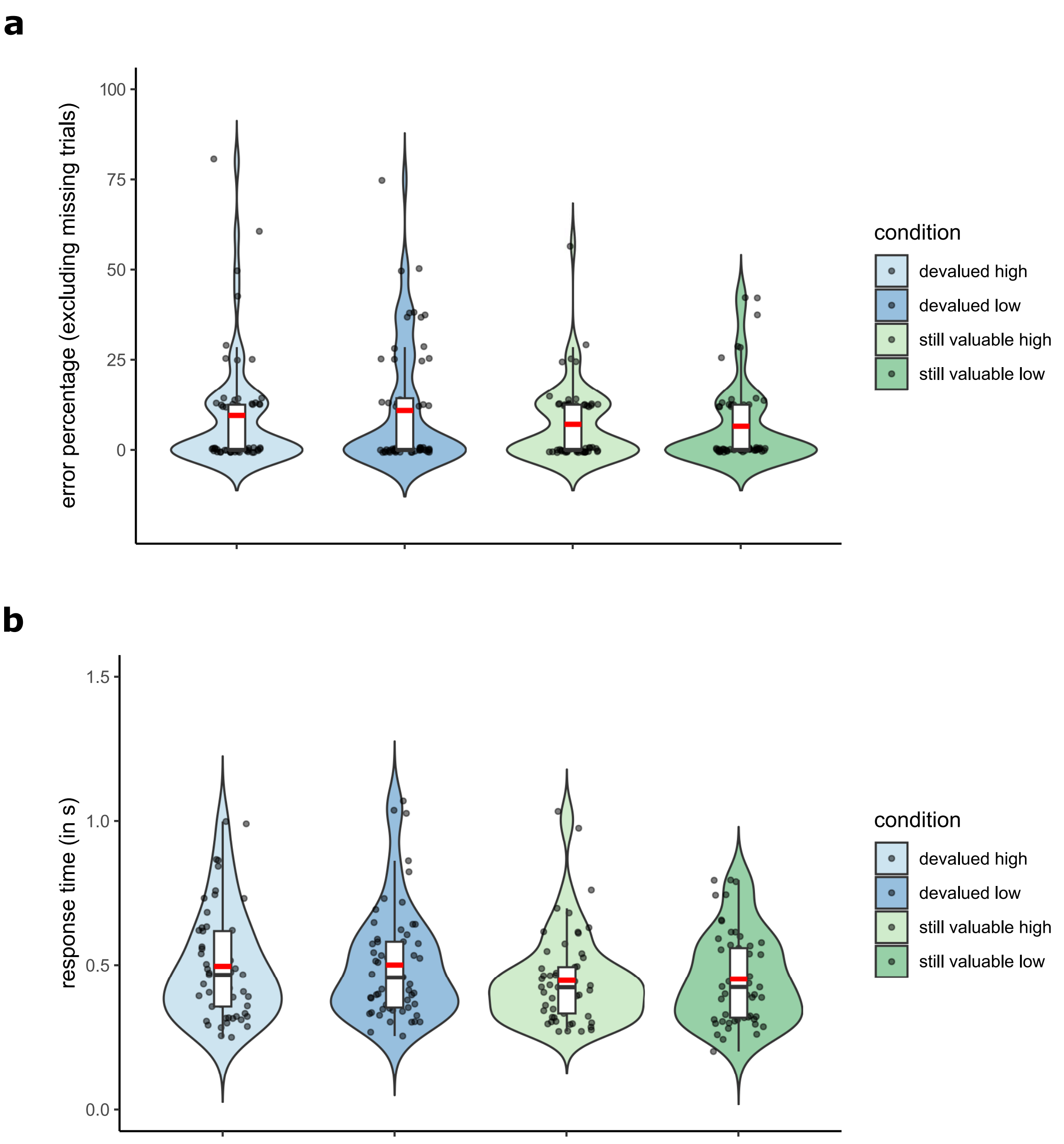

Figure S2: a: Distribution of mean error percentage in devaluation blocks according to value and reward magnitude; b: distribution of mean response time in correct trials according to value and reward magnitude. Conditions: devalued = trials with devalued outcome, still valuable = trials in devaluation blocks with still valuable outcome, high = associated with high reward magnitude during training, low = associated with low reward magnitude during training. Black vertical lines represent median values, and red vertical lines represent mean values. N = 57.

Distribution of Differences in Error Percentage and RTs Between Devalued and Still Valuable Trials

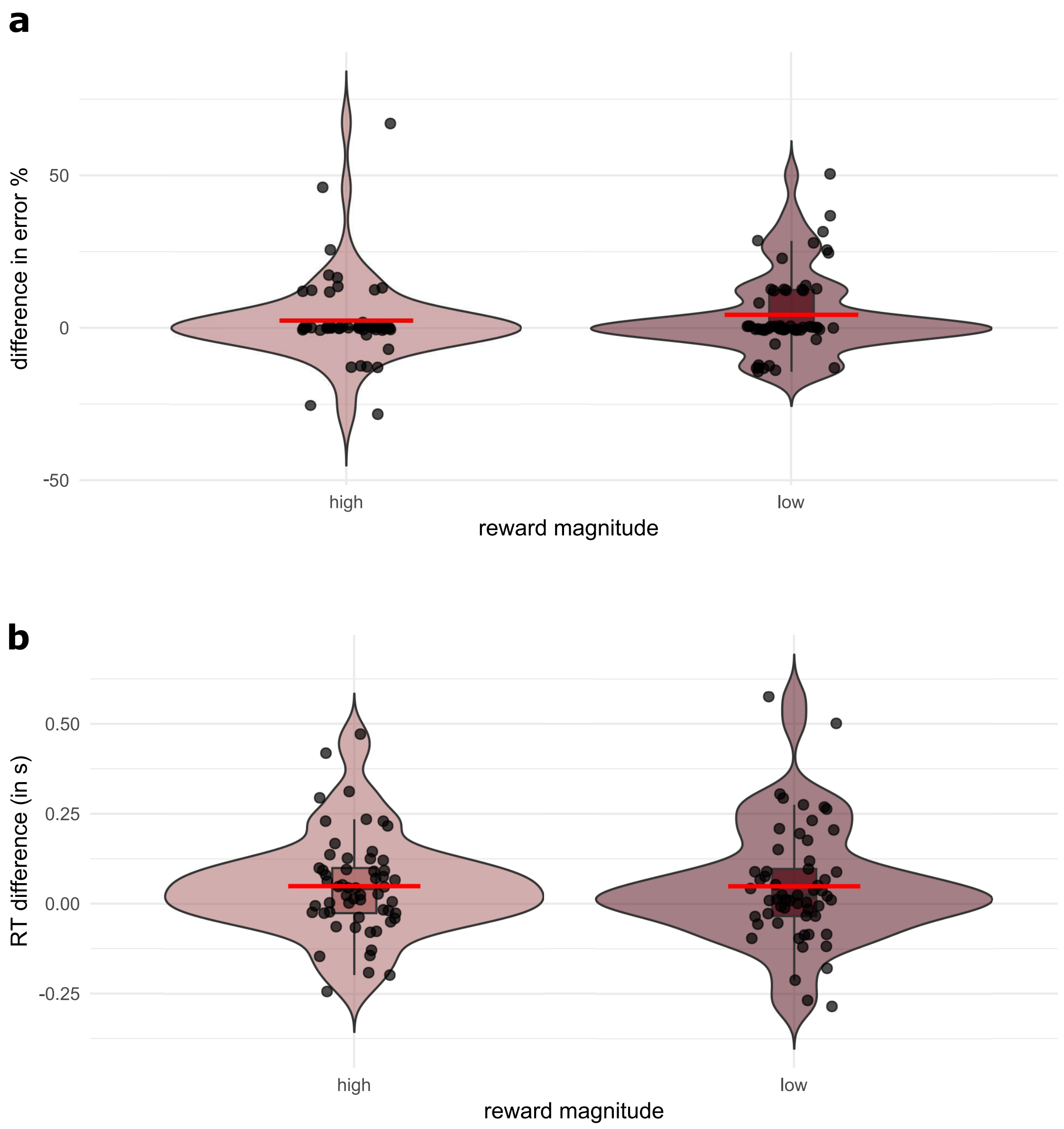

Figure S3: a: Distribution of the mean difference in error percentage between trials with devalued and still valuable outcome according to reward magnitude; b: distribution of the difference in mean response time in correct trials between trials with devalued and still valuable outcome according to reward magnitude. Conditions: high = associated with high reward magnitude during training, low = associated with low reward magnitude during training. Red vertical lines represent mean values. N = 57.

Devaluation Performance per Block

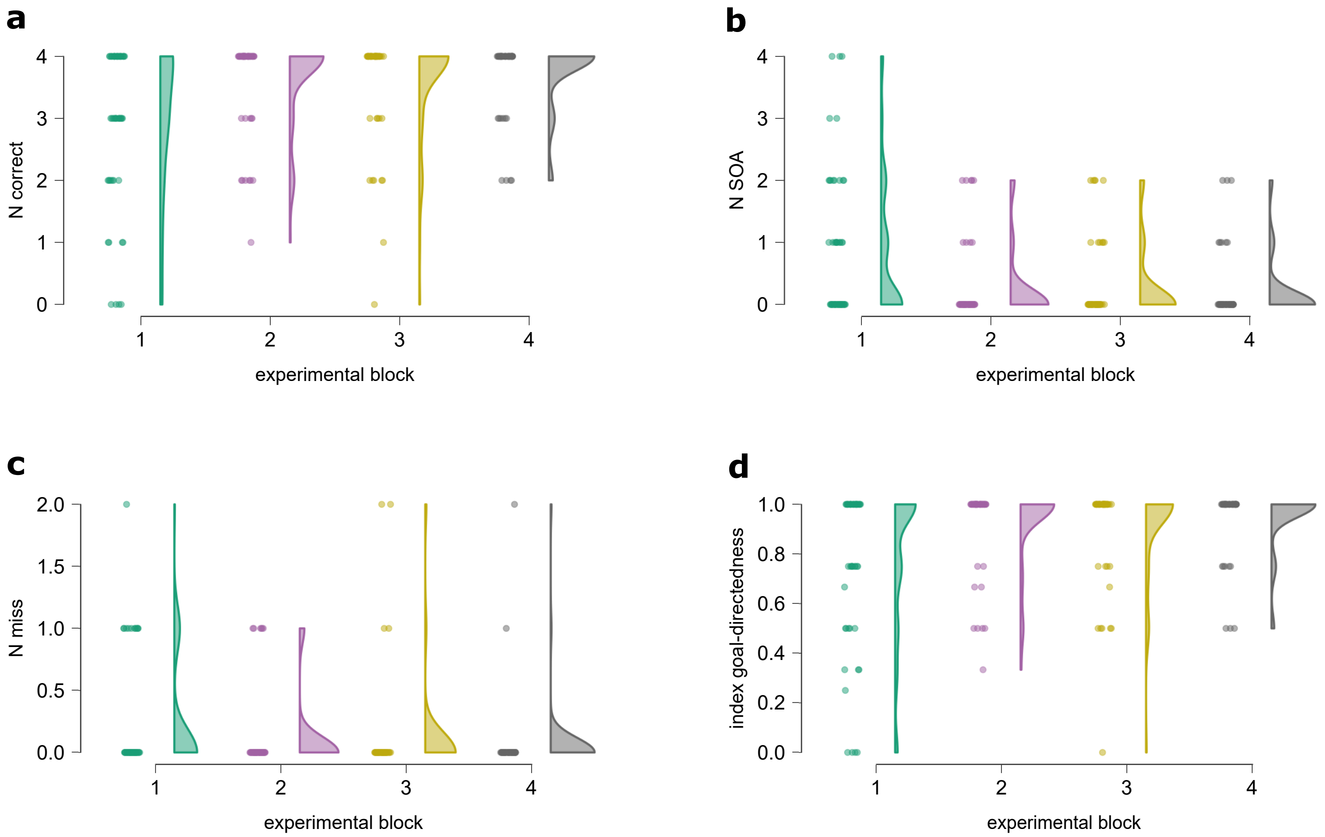

Figure S4: Behavioral performance in trials with devalued outcomes across experimental blocks. a: Number of correct adjustments to devalued outcomes; b: number of slips-of-action (SOA); c: number of missing responses; d: index of goal-directed behavior computed separately per block as $N_{correct} \div (N_{correct} + N_{SOA})$.

Results Univariate Analysis

Label (peak)	Peak [x, y, z]	Cluster size	P (FWE-corr. at cluster level)
**Contrast: correct adjustment to devaluation > baseline**			
**Insula R**	[30, 28, 0]	9377	$< .001$
Putamen R	[18, 14, -2]		
Insula L	[-32, 22, 0]		
**Frontal Supp. R**	[26, -2, 58]	5695	$< .001$
Frontal Mid L	[-24, 2, 62]		
Supp. Motor L	[-8, 24, 48]		
**Occipital Mid R**	[38, -76, 36]	7095	$< .001$
Precuneus R	[10, -68, 56]		
Precuneus L	[-10, -64, 58]		
**Supramarginal R**	[42, -36, 44]	790	$< .001$
Parietal Inf. R	[38, -46, 52]		
Parietal Inf. R	[46, -38, 58]		
**Cerebellum Crus1 L**	[-38, -48, -32]	333	$< .001$
Cerebellum Crus1 L	[-30, -62, -30]		
**Cerebellum 6 R**	[30, -60, -30]	367	$< .001$
**Calcarine R**	[12, -92, 0]	1261	$< .001$
Fusiform R	[28, -76, -12]		
Lingual R	[14, -86, -8]		
**Lingual L**	[-10, -94, -6]	378	$< .001$
Occipital Sup. L	[-12, -96, 12]		
**Frontal Mid R**	[42, 34, 36]	528	$< .001$
Frontal Inf. Tri. R	[46, 32, 28]		
Frontal Inf. Tri. R	[44, 36, 18]		
**Fusiform L**	[-32, -78, -12]	139	$.04$
**Frontal Inf. Oper. R**	[54, 10, 28]	172	$.01$
**Temporal Inf. L**	[-58, -44, -12]	235	$< .01$
Temporal Inf. L	[-54, -62, -8]		
Temporal Inf. L	[-52, -54, -12]		
**Hippocampus L**	[-24, -38, 2]	164	$.02$
Fusiform L	[-26, -58, -12]		
Fusiform L	[-22, -44, -10]		

Table S1: Brain areas showing significant positive activation in trials where behavioral responses were correctly adjusted to devalued outcomes vs. implicit baseline.
